# The *Epichloë* *festucae* Antifungal Protein *Efe*-AfpA Is also a Possible Effector Protein Required for the Interaction of the Fungus with Its Host Grass *Festuca rubra* subsp. *rubra*

**DOI:** 10.3390/microorganisms9010140

**Published:** 2021-01-09

**Authors:** Ruying Wang, Simin Luo, Bruce B. Clarke, Faith C. Belanger

**Affiliations:** 1 Department of Plant Biology, Rutgers University, New Brunswick, NJ 08901, USA; ruying.wang@oregonstate.edu (R.W.); 2018120011@njau.edu.cn (S.L.); bruce.clarke@rutgers.edu (B.B.C.); 2 Department of Horticulture, Oregon State University, Corvallis, OR 97331, USA; 3 College of Agro-Grassland Science, Nanjing Agricultural University, Nanjing 210095, China

**Keywords:** antifungal protein, endophyte, strong creeping red fescue, symbiosis

## Abstract

Strong creeping red fescue (*Festuca rubra* subsp. *rubra*) is a commercially important low-maintenance turfgrass and is often naturally infected with the fungal endophyte *Epichloë* *festucae*. *Epichloë* spp. are endophytes of several cool-season grass species, often conferring insect resistance to the grass hosts due to the production of toxic alkaloids. In addition to insect resistance, a unique feature of the strong creeping red fescue/*E. festucae* symbiosis is the endophyte-mediated disease resistance to the fungal pathogen *Clarireedia jacksonii,* the causal agent of dollar spot disease. Such disease resistance is not a general feature of other grass/ *Epichloë* interactions. *E. festucae* isolates infecting red fescue have an antifungal protein gene *Efe-afpA*, whereas most other *Epichloë* spp. do not have a similar gene. The uniqueness of this gene suggests it may, therefore, be a component of the unique disease resistance seen in endophyte-infected red fescue. Here, we report the generation of CRISPR-Cas9 *Efe-afpA* gene knockouts with the goal of determining if absence of the protein in endophyte-infected *Festuca rubra* leads to disease susceptibility. However, it was not possible to infect plants with the knockout isolates, although infection was possible with the wild type *E. festucae* and with complemented isolates. This raises the interesting possibility that, in addition to having antifungal activity, the protein is required for the symbiotic interaction. The antifungal protein is a small secreted protein with high expression *in planta* relative to its expression in culture, all characteristics consistent with effector proteins. If *Efe-*AfpA is an effector protein it must be specific to certain interactions, since most *Epichloë* spp. do not have such a gene in their genomes.

## 1. Introduction

Strong creeping red fescue (*Festuca rubra* subsp. *rubra*) is a commercially important low-maintenance turfgrass and is often naturally infected with the fungal endophyte *Epichloë festucae* [1,2]. *Epichloë* spp. are endophytes of several cool-season grass species, often conferring insect resistance to the grass hosts due to the production of toxic alkaloids [1,2]. For turfgrasses, cultivars containing *Epichloë* endophytes are desired because of the enhanced insect resistance [3]. In addition to insect resistance, a unique feature of the strong creeping red fescue/*E. festucae* symbiosis is the endophyte-mediated disease resistance to the fungal pathogen *Clarireedia jacksonii* (formerly *Sclerotinia homoeocarpa*) [4], the causal agent of dollar spot disease [5]. Such endophyte-mediated disease resistance is not seen in the cultivated grasses ryegrass (*Lolium perenne* L.) or tall fescue (*Lolium arundinaceum*) [4,6]. As discussed previously [6], there is conflicting literature on *Epichloë* spp. mediated disease resistance in various grass hosts but no field-level resistance has been reported other than for the fine fescues [4].

The mechanism underlying the unique endophyte-mediated disease resistance in strong creeping red fescue is not yet established. We are pursuing the possibility that it may be due to the presence of an abundant secreted antifungal protein produced by the fungal endophyte *E. festucae*. In a previous quantitative transcriptome study of the *E. festucae*–strong creeping red fescue interaction, the second most abundant fungal transcript was found to encode a small (6278 Daltons) secreted protein similar to antifungal proteins from *Penicillium* and *Aspergillus* species [7]. The purified *E. festucae* antifungal protein, *Efe*-AfpA, was shown to inhibit the growth of *C. jacksonii* and to cause membrane damage in plate assays [6]. The antifungal protein gene, *Efe-afpA* (gene model EfM3.0636600, http://csbio-l.csr.uky.edu/endophyte/; GenBank accession MG925781.1) [1] found in *E. festucae* infecting strong creeping red fescue is not present in most *Epichloë* genomes for which whole genome sequence is available, being present only in *E. baconii*, *E. aotearoae*, and *E. inebrians* [6,7]. The antifungal activity, transcript abundance and the limited existence of the gene among *Epichloë* spp. suggested the *E. festucae* antifungal protein may be a component of the unique endophyte-mediated disease resistance observed in strong creeping red fescue. If so, this protein may have the potential to be utilized as a biological control agent/product for the suppression of dollar spot or other diseases on fine fescues and other important turfgrasses.

A common approach to evaluate the role of a particular protein in a physiological process is to generate a gene knockout, in which the target gene is inactivated, and evaluate the phenotype of the mutant. In *Epichloë* spp., knockouts of numerous genes have been reported using the approach of homologous recombination to replace the target gene with a selectable marker [8]. More recently, CRISPR-Cas9 technology has emerged as an efficient approach to targeted gene deletions [9]. With this method, single guide RNA (sgRNA) with a 20 bp target sequence directs the Cas9 endonuclease to precisely generate double strand breaks. Repairing the breaks by nonhomologous end joining is likely to introduce mistakes causing frame shifting, which frequently inactivates the gene of interest. CRISPR-Cas9 technology has been established for animal, plant, yeast, and filamentous fungal systems [10,11,12,13]. Here, we report the application of CRISPR-Cas9 gene deletion technology to the *E. festucae* antifungal protein gene. Two independent gene knockout isolates were obtained but they were unable to infect strong creeping red fescue plants suggesting that *Efe-afpA* may be essential for infection and association with the host.

## 2. Materials and Methods

### 2.1. Construction of Vectors Used for the Knockout and Rescue Transformations

For vector construction, genomic DNA of the Rose City isolate of *E. festucae* [14] was extracted from cultures grown on potato dextrose agar or in potato dextrose broth (Difco Laboratories, Detroit, MI, USA) using the method described by Moy et al. [15]. The details of the construction of the vector used for homologous recombination are described in Wang [16].

Two CRISPR-Cas9 vectors were constructed. The vector p-*hph*-P*tef1-cas9*-pksP-gRNA [17], (obtained from the Fungal Genetics Stock Center, Manhattan, KS, USA), which contains the *hph* resistance gene, the human codon-optimized *cas9* gene with the SV40 nuclear localization sequence, and gRNA targeting the *Aspergillus fumigatus* polyketide synthase gene *pks*P, was modified to have gRNA targeting *Efe-afpA*. The *Efe-afpA* gRNA was designed to target the sequence 5ʹ-GGCATTCTGATCACGTATGA-3′ immediately upstream of the protospacer-adjacent motif (PAM) sequence AGG in the first exon of *Efe*-*afpA*. Primers AFP-Del2-F and AFP-Del2-R (Table S1) were used to change the existing gRNA sequence of p-*hph*-P*tef1-cas9*-pksP-gRNA to the *Efe-afpA* gRNA target sequence with the Q5 Site-Directed Mutagenesis Kit (New England BioLabs Inc., Ipswich, MA, USA). Since p-*hph*-P*tef1-cas9*-pksP-gRNA is a large plasmid (>13 kb), PrimeSTAR HS Premix (TaKaRa Bio Inc., Shiga, Japan) was used instead of Q5 Hot Start High-Fidelity 2X Master Mix in the Q5 Site-Directed Mutagenesis Kit. PCR was performed in a 25 µL volume with one ng of the vector p-*hph*-P*tef1-cas9*-pksP-gRNA, 0.3 µM of each forward and reverse primer (Integrated DNA Technologies, Inc., Coralville, IA, USA) and 12.5 µL of PrimeSTAR HS Premix. Two-step PCR was performed by template denaturation at 98 °C for 10 s followed by 6 min extension at 68 °C for 30 cycles. The PCR product was used in the remaining steps following the Q5 Site-Directed Mutagenesis Kit manufacturer’s protocol. The resulting plasmid was designated pD2P6.

A second CRISPR-Cas9 vector containing a *Trichoderma reesei* codon-optimized *cas9* with a SV40 nuclear localization sequence was constructed utilizing some of the components of pD2P6 described above and the *cas9* sequence from plasmid pDHt/sk-PC [18], (provided by Gen Zou and Zhihua Zhou). In this construct, the *T. reesei* codon-optimized *cas9* gene includes the *T. reesei* constitutive pyruvate decarboxylase promoter (Ppdc) and terminator (Tpdc) sequences. The new plasmid was assembled from three fragments. Fragments 1 and 2 were amplified from pD2P6 with primers sets BG2F/FullerGR and FullerGF2/PCseqR1, respectively. The Ppdc-*T. reesei* codon-optimized *cas9-*Tpdc fragment from the plasmid pDHt/sk-PC was amplified using primers GTCas9F and GTCas9R to also overlap fragments 1 and 2. The 100 μL reactions contained 2X Phusion Green Hot Start II High-Fidelity PCR Master Mix (Thermo Fisher Scientific, Waltham, MA, USA), 0.5 µM of each oligonucleotide, and either 125 pg of pD2P6 or 2 ng of pDHt/sk-PC as template. The PCR reaction conditions for fragment 1 and 2 were an initial denaturation step at 98 °C for 30 s, followed by 30 cycles of 10 s denaturation at 98 °C, 30 s annealing at 60 °C, and 5 min extension at 72 °C. The PCR reaction conditions for amplification of the Ppdc-toCas9-Tpdc fragment were an initial denaturation step at 98 °C for 30 s, followed by 30 cycles of 10 s denaturation at 98 °C, 30 s annealing at 65 °C, and 3.5 min extension at 72 °C. PCR products were then purified by using 0.7X Agencourt AMPure XP (Beckman Coulter, Brea, CA, USA) and assembled with Gibson Assembly Master Mix (E2611S; New England BioLabs Inc., Ipswich, MA, USA). In a 20 μL reaction, 0.11, 0.10, and 0.06 pmol of each fragment 1, 2, and Ppdc-toCas9-Tpdc, respectively, were mixed with 2X Gibson Assembly Master Mix and incubated at 50 °C for 1 h. One μL of the assembly reaction was used to transform 25 μL NEB 10-beta Electrocompetent *E. coli* cells (New England BioLabs Inc., Ipswich, MA, USA). The resulting plasmid was designated pG4.

### 2.2. Construction of a Plasmid for Complementation of the Efe-afpA Knockouts

A PCR fragment of the *Efe-afpA* gene, including 596 bp upstream and 573 bp downstream of the coding sequence, was cloned into the pMiniT 2.0 vector according to the instructions provided with the NEB PCR Cloning Kit (New England Biolabs, Ipswich, MA, USA). The PCR fragment was generated from *E. festucae* genomic DNA by using primers 60F and 60R. The primer sequences include 20 and 21 bp homologous to the 5′ and 3′ upstream and downstream sequences of the *Efe-afpA* gene, respectively, and 40 bp of tag sequences not found in the *E. festucae* genome. The nonhomologous sequences provided sequences that could be used as primers for screening transformants for successful integration of the intact *Efe-afpA* gene PCR fragment.

### 2.3. Protoplast Preparation

Protoplast preparation and transformation of the wild type Rose City isolate and the two knockout isolates were modified from the procedures described by Turgeon et al. [19] and Sonderegger et al. [20]. The fungal isolates were grown in 50 mL potato dextrose broth medium in 200 mL flasks for two weeks at room temperature with shaking at 80 rpm. The fungal mycelium from two flasks was collected in 50 mL tubes by centrifugation at 5000 rpm for 10 min at 4 °C in a Sorvall Legend X1R centrifuge. The supernatant was discarded and the mycelium was transferred to sterile paper towels and blotted dry. The mycelium was then distributed to four 50 mL centrifuge tubes in 0.8–1 g aliquots. A solution of lysing enzymes from *Trichoderma harzianum* (Item L1412, Sigma-Aldrich, St. Louis, MO, USA) was prepared by dissolving the enzyme powder in 5 mL lysis buffer (50 mM phosphate buffer, pH 5.8, 0.7 M KCl). After the powder was dissolved, an additional 10 mL of lysis buffer was added, and the solution was filter sterilized. The sterilized enzyme solution was then distributed evenly to the four tubes of fungal mycelium and the volume in each tube was brought to 10 mL with lysis buffer. The samples were incubated at 30 °C with gentle shaking (50 rpm) for 18 h. The protoplasts were separated from intact mycelium and cell wall debris by filtering the suspension through four layers of sterile cheesecloth. The protoplasts were pelleted by centrifugation at 5000 rpm for 5 min at 4 °C in a SS-34 fixed angle rotor. The protoplast pellets from the four tubes were resuspended in a total volume of 10 mL 0.7 M KCl and combined into one tube and were centrifuged again as before. The protoplast pellet was then washed three times with 10 mL STC buffer (1.2 M sorbitol, 10 mM Tris-HCl, pH 7.5, 50 mM CaCl_2_ H_2_O), pelleting the cells between washes as before. The final protoplast pellet was resuspended in 100 μL STC buffer. The protoplasts were counted using a hemocytometer and adjusted to a density of approximately 10^8^ mL^−1^ with STC. 

### 2.4. Fungal Transformation and Screening of Transformants

All transformations used the polyethylene glycol-mediated protoplast transformation method as described previously [19]. For the unsuccessful CRISPR-Cas9 knockout, 10 μg of plasmid pD2P6 in 25 μL was used. For the CRISPR-Cas9 knockout, 10 μg of in vitro transcribed gRNA and 10 μg of pG4 plasmid in a total of 25 μL were co-transformed into approximately 6 × 10^6^ *E*. *festucae* Rose City protoplasts. Single-guide RNA was generated in vitro using GeneArt Precision gRNA Synthesis Kit (Thermo Fisher Scientific, Waltham, MA, USA) according to the manufacturer’s instructions with primers gF2 and gR2.

For complementation, protoplasts of the two knockout isolates were co-transformed with 2–5 μg of plasmid FB009 (Addgene plasmid #119707, Watertown, MA, USA) containing the selectable marker for the antibiotic G418 and either 2.5 μg of a PCR fragment containing the *Efe-afpA* gene or 5 μg of the complementation plasmid described above.

Transformed protoplasts were plated into molten regeneration medium as described by Turgeon et al. [19] and incubated overnight at 30 °C. The plates were then overlaid with 10 mL of PDA containing 150 μg mL^−1^ hygromycin for the knockouts or 200 μg mL^−1^ G418 for the rescue transformants. Transformed fungal colonies that emerged through the antibiotic layer were grown on PDA plates containing the appropriate antibiotic overlaid with cellophane.

For screening the transformants fungal genomic DNA was extracted by using the DNeasy Plant Pro Kit (Qiagen, Germantown, MD, USA). Knockout transformants were identified by sequencing PCR products amplified from the *Efe-afpA* genomic region with primers cafp-f and AFPr. The rescue transformants were identified by sequencing PCR products amplified with the primers COM1F and COM1R. The PCR reaction conditions were one cycle at 98 °C for 30 s; 30 cycles at 98 °C for 10 s, 60 °C for 30 s, and 72 °C for 30 s; and one cycle at 72 °C for 5 min. PCR products were sequenced directly. For each sequencing reaction, a 5 μL aliquot of each PCR product was treated with 2 µL ExoSAP-IT (USB Corp., Cleveland, OH, USA) to remove unincorporated primers and excess dNTPs. The ExoSAP-IT reaction was performed at 37 °C for 15 min followed by heating at 80 °C for 15 min to inactivate the enzymes, and then sequenced (Genewiz, Inc., South Plainfield, NJ, USA). Single spore isolates of the CRISPR-Cas9 knockout mutants and the rescue isolates were generated by washing a plate of the knockout and rescue isolates with sterile water and plating dilutions of the water containing spores onto new plates. The status of isolated colonies originating from single spores was confirmed by sequencing PCR fragments as described for the primary screening.

### 2.5. Strong Creeping Red Fescue Inoculation with E. festucae Isolates

The knockout and rescue isolates and the wild type Rose City isolate were used to inoculate germinating endophyte-free seedlings of the strong creeping red fescue cultivar Kent using a modification of the method described by Latch and Christensen [21]. Seeds were sterilized as described by Johnson-Cicalese et al. [22] and germinated in the dark on 3% (*w*/*v*) water agar in 10 cm square petri dishes. The petri dishes were maintained in an upright position to allow the seedlings to grow gravitropically. After five days in the dark, they were put under light for two days. The seed coats were removed aseptically and a 10 μL aliquot of potato dextrose broth was applied to the crown area of each seedling. A small piece of fungal tissue from the edge of a colony growing on potato dextrose agar was homogenized in 1 mL sterile water in a mortar and a 5 μL aliquot of the homogenate was applied to the crown of each seedling on top of the potato dextrose broth. After about eight days, fungal hyphae could be seen growing around the seedling. At that point, the seedling was wounded in the crown region with a 30-gauge needle (BD Ultra-Fine Insulin Syringes, Franklin Lakes, NJ, USA) to allow the growing fungal hyphae to enter the plant. Three to five days after wounding the seedlings were transferred to Fafard Canadian Grow Mix 2 (Agawam, MA, USA). The transplanted seedlings were acclimated in the lab at room temperature for several weeks and then transferred to the greenhouse. After several new tillers had emerged, the inoculated plants were screened for endophyte infection by an immunoblot assay (Phytoscreen, Agrinostics, Ltd. Co., Watkinsville, GA, USA) and positive plants were confirmed microscopically. For microscopic analysis, leaves or leaf sheaths were pretreated by submerging tissue in 95% ethanol in 24-well microtiter plates for more than 4 h to remove chlorophyll. They were then incubated in aniline blue—lactic acid stain [23] for 15 min at 40 °C and examined by microscopy for intercellular fungal hyphae.

Inoculation with the complemented knockout isolates was further confirmed by generating PCR fragments with primers COM1F and RescueR from DNA isolated from the inoculated plants. The PCR fragment was sequenced confirming the fragment was from *Efe-afpA*. The COM1F sequence is part of the 60F primer used to amplify the *Efe-afpA* gene for complementation of the knockout isolates. This serves as a tag for the complementation sequence since it is not normally present in the *E. festucae* genome.

### 2.6. PacBio Long Read Sequencing

Genomic DNA of the wild type *E. festucae* Rose City isolate was isolated as described by Dellaporta et al. [24]. Library preparation and sequencing was done by Genewiz (South Plainfield, NJ, USA). PacBio SMRTbell libraries were prepared per the manufacturer’s instructions and sequenced on the PacBio Sequel platform with v3.0 chemistry. The genome sequence of the wild type Rose City isolate was de novo assembled by Genewiz using HGAP4 software with default parameters, generating 46 contigs. This Whole Genome Shotgun project has been deposited at DDBJ/ENA/GenBank under the accession JADWOS000000000. The version described in this paper is version JADWOS010000000. The BioProject ID is PRJNA677971.

### 2.7. Quantitative RT-PCR

Total RNA was isolated from frozen mycelium of the *E. festucae* Rose City isolate and from leaf sheath tissue of the Rose City isolate-infected plant tissue. The tissues were homogenized in a HT Mini bead beater (Ops Diagnostics, Lebanon, NJ, USA) and RNA extracted using the ZR Fungal/Bacterial RNA MiniPrep Kit (Zymo Research, Irvine, CA, USA) according to the manufacturer’s instructions. A TURBO DNA-free kit (Invitrogen, Carlsbad, CA, USA) was used to remove any genomic DNA in the RNA samples. cDNA synthesis was performed on 1.5 μg RNA using the High-Capacity cDNA Reverse Transcription Kit (Applied Biosystems, Foster City, CA, USA). Quantitative RT-PCR was performed using the Power SYBR Green PCR Master Mix (Applied Biosystems) on a StepOnePlus RT-PCR system (Applied Biosystems) with three technical replicates per sample. *Efe-afpA* transcripts were normalized to the expression levels of elongation factor 2 (EfM3.021210) and 40S ribosomal protein S22 (EfM3.016650) using primers described by Chujo and Scott [25] and the 2^−ΔΔCt^ method as described by Livak and Schmittgen [26]. Primer sequences used for quantitative RT-PCR are given in Table S1.

## 3. Results

### 3.1. The Homologous Recombination Approach Was Unsuccessful In Generating a Gene Deletion of the E. festucae Antifungal Protein Gene

The first attempt at generating a knockout of the antifungal protein gene used the well-established homologous recombination method, which has been successful for many *Epichloë* genes [8]. In this method, a plasmid for transformation is constructed in which sequences identical to the region of the targeted gene flank an antibiotic resistance gene. When such a plasmid is transformed into protoplasts of the fungus, homologous recombination occurs at the sites of the flanking sequences and the antibiotic resistance gene replaces the targeted gene generating the gene knockout. Filamentous fungi typically require long flanking sequences for efficient homologous recombination to occur. Generally flanking sequences of 1.5 to 2.5 kb are used and the frequencies of gene knockouts range from 1–25% [8].

The transformation vector for the homologous recombination method was made by ligating the hygromycin phosphotransferase (*hph*) cassette to 598 bp left flank and 634 bp right flank of the antifungal protein gene amplified from *E. festucae* genomic DNA. The antifungal protein gene is a small gene surrounded by A/T rich repeated sequences, which made it impossible to use longer unique flanking sequences for this approach. Over 250 hygromycin resistant transformants from the homologous recombination method were screened by PCR but none were the desired gene replacement mutant. Although, the homologous recombination approach is widely used in *Epichloë* species, this conventional fungal gene knockout method was unsuccessful in our study. These results are likely because only short unique upstream and downstream regions were available for vector construction since the antifungal protein gene is flanked on both sides by repeated sequences.

### 3.2. Efe-afpA Knockouts Recovered by Using CRISPR-Cas9

The CRISPR-Cas9 technology has recently become the method of choice for gene knockouts in many organisms because it requires only 20 bases of sequence identity to the target gene [27]. The first attempt at generating an *Efe-afpA* gene knockout using the CRISPR-Cas9 approach used a construct that was successfully used in *Aspergillus fumigatus* and which had a Cas9 enzyme sequence that was codon-optimized for use in human cells [17]. However, that construct was not successful in generating a knockout in *E. festucae*.

Liu et al. [18] reported that the human codon-optimized *cas9* gene did not function in *Trichoderma reesei* but that a construct with a *Trichoderma* codon-optimized *cas9* gene did. Phylogenetically, *Epichloë* is more closely related to *Trichoderma* than it is to *Aspergillus*. Both *Epichloë* and *Trichoderma* are genera in the order Hypocreales within the class Sordariomycetes, whereas *Aspergillus* is in the order Eurotiales within the class Eurotiomycetes. We, therefore, modified the first CRISPR-Cas9 construct to incorporate the *T. reesei* codon-optimized *cas9* gene rather than the human codon-optimized gene. The modified construct was successful in generating *Efe-afpA* gene knockouts.

**Figure 1:**
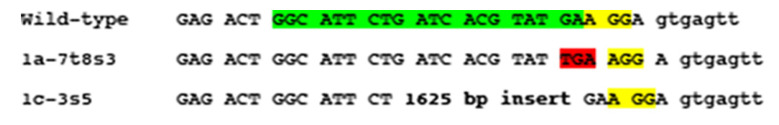
Sequence alignment of part of the first exon of *Efe-afpA* of the wild-type Rose City isolate of *E. festucae*, and the two knockout mutants. A single base pair insertion (T) in 1a-7t8s3 introduced a stop codon, highlighted in red. Knockout isolate 1c-3s5 had a 10 bp deletion of *Efe-afpA* and a 1625 bp insertion, which was a fragment from the transformation vector pG4. The target guide RNA sequence and PAM sequence designed for CRISPR-Cas9 are highlighted in green and yellow, respectively. Upper case letters indicate *Efe-afpA* coding sequence and lower case letters indicate intron sequence.

To knock out *Efe-afpA* the sgRNA was designed to target a sequence in the first exon of the gene. The Cas9 endonuclease generated double-strand breaks at the target site of the antifungal protein gene, and mutations were introduced when repairing the break. After screening 42 hygromycin resistant isolates, two *Efe-afpA* knockout isolates were identified. One, 1a-7t8s3, had a single T insertion at the Cas9 target site, which immediately introduced an early stop codon (Figure 1). The second knockout isolate, 1c-3s5, had a 10 bp deletion of the *Efe-afpA* gene coupled with an insertion of 1625 bp at the target site, which was identified as a fragment from the transformation vector (Figure 1 and Supplemental Figure S1).

The two knockout isolates were complemented by transformation with the intact *Efe-afpA* gene including 596 bp 5′ of the coding sequence and 573 bp 3′ of the coding sequence. Two independent complemented isolates of each **Δ***Efe*-*afpA* isolate were recovered, designated 1a-7t8s3-B, 1a-7t8s3-I, 1c-3s5-X, and 1c-3s5-L.

**Figure 2:**
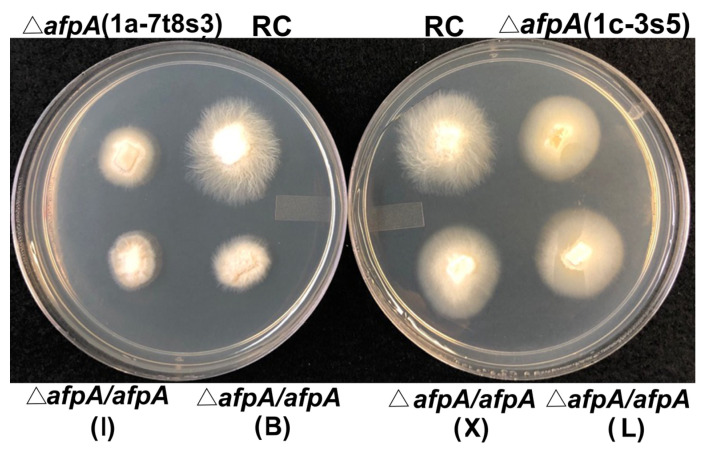
Comparison of colony morphology of the wild type *E. festucae* Rose City (RC) isolate, the two knockout isolates, and the four independent complemented knockout isolates on potato dextrose agar plates. The letter designations of the four complemented knockout isolates (I, B, X, and L) are given in parentheses.

Both of the knockout isolates exhibited slightly slower growth rates on potato dextrose agar than the wild type Rose City isolate, which was not fully restored in the complemented isolates (Figure 2). Microscopic observation of the hyphae of the knockout and complemented isolates did not reveal any differences with the wild type.

### 3.3. Efe-afpA Knockout Isolates Were Unable to Infect Strong Creeping Red Fescue

The reason for generating the *Efe-afpA* knockouts was to determine the effect of the fungus lacking *Efe-afpA* on the dollar spot susceptibility of strong creeping red fescue by inoculating uninfected plants with the knockout isolates and comparing disease levels with the wild type endophyte-infected plants. No successful inoculations of the knockout isolates were obtained, precluding such a comparison. The inoculation method used was successful in inoculating strong creeping red fescue seedlings with the wild type Rose City isolate of *E. festucae,* as well as the complemented knockout isolates (Table 1). However, the knockout isolates appeared unable to infect strong creeping red fescue seedlings. The Δ*Efe*-*afpA* knockout isolates apparently have reduced ability to infect their host grass. That neither of the two independent Δ*Efe*-*afpA* isolates was able to infect the strong creeping red fescue seedlings and that the wild type and three of the four independent complemented isolates were able to infect suggests that *Efe-*AfpA may have a role in the ability of the fungus to infect and establish a symbiotic association with the plant. 

### 3.4. Comparison of Expression Levels of Efe-afpA In Culture and In Planta

**Figure 3:**
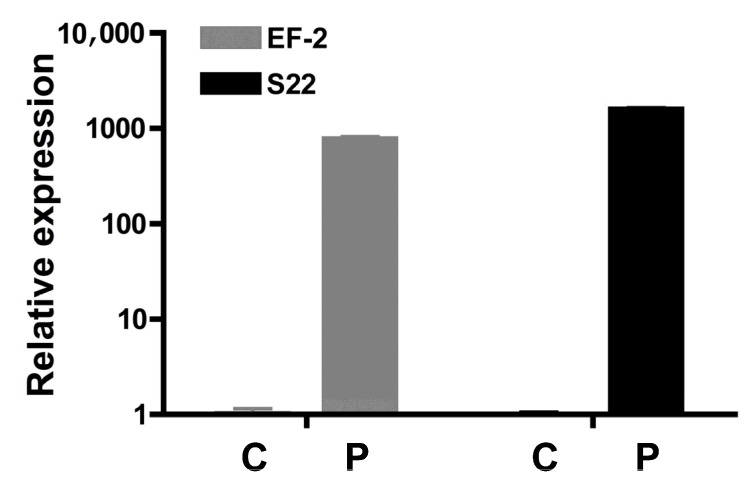
Comparison of expression level of *Efe-afpA* in culture (C) with expression *in planta* (P) relative to expression levels of two reference genes, elongation factor 2 (EF-2; gene model EfM3.021210) and 40S ribosomal protein S22 (S22; gene model EfM3.016650). The y-axis is a logarithmic scale.

The inoculation data described above indicated that *Efe-afpA* may be required for the symbiotic association with the host, suggesting that *Efe-AfpA* may function as an effector protein (discussed more below). One general characteristic of fungal effector proteins is that they are more highly expressed in association with their hosts than in culture [28]. Quantitative RT-PCR revealed that the expression level of *Efe-afpA* is over 700-fold higher *in planta* than in culture when compared with two reference genes (Figure 3). 

### 3.5. Identification of Genomic Region of Efe-afpA in the E. festucae Rose City Isolate

**Figure 4:**
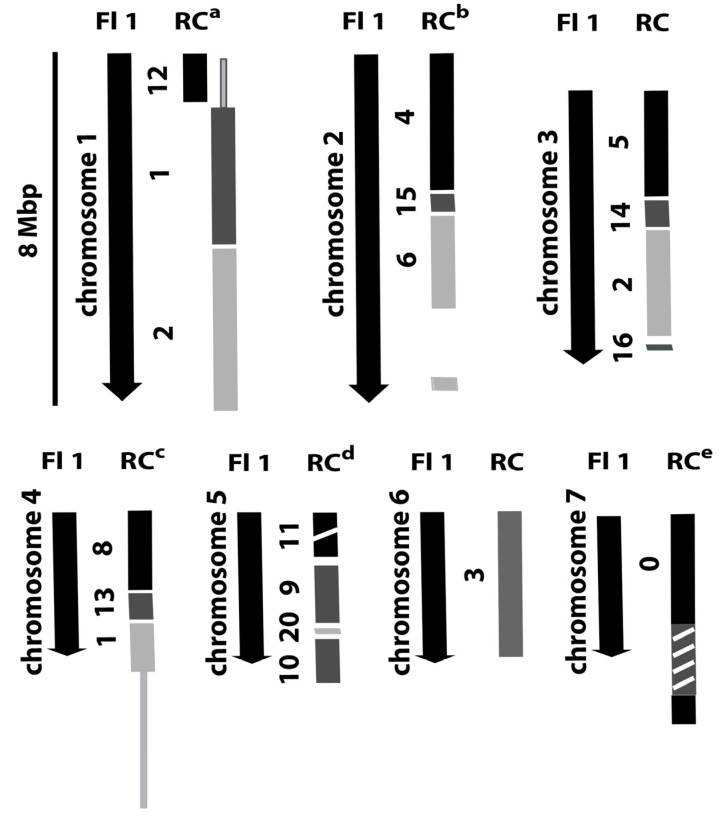
Diagram of relationships of the *E. festucae* Rose City (RC) isolate genome sequence contigs to the chromosomes of the *E. festucae* Fl1 isolate. ^a^ Line extending from RC contig 1 indicates region of contig 1 that is homologous to Fl1 chromosome 4. ^b^ Gap in RC contig 6 indicates the corresponding region of Fl1 that is found in RC contig 0. ^c^ Line extending from RC contig 1 indicates region of contig 1 that is homologous to Fl1 chromosome 1. ^d^ Break in RC contig 11 indicates an approximately 400,000 bp insert relative to Fl1 chromosome 5. ^e^ Hashed box in RC contig 0 indicates region of contig homologous to Fl1 chromosome 2.

As previously reported, *Efe-afpA* is the single gene on a 21.4 kb genome sequence contig from the *E. festucae* 2368 isolate and is flanked by repeated sequences [1,6, http://csbio-l.csr.uky.edu/endophyte/]. To learn more of the genomic context of *Efe-afpA*, we generated a PacBio long read sequence for the wild type Rose City *E. festucae* isolate. The characteristics of the sequencing are summarized in Table 2. The sequences were de novo assembled and resulted in 46 contigs, 19 of which could be aligned to the 7 chromosomes of the finished genome sequence of the *E. festucae* Fl1 isolate, an endophyte of *Festuca trachyphylla* (hard fescue) [29]. The remaining 27 contigs were matches to the *E. festucae* Fl1 isolate mitochondrion sequence. Figure 4 depicts the correspondence of the *E. festucae* Rose City isolate contigs with the 7 chromosomes of the *E. festucae* Fl1 isolate. As expected for these two *E. festucae* isolates, there is overall correspondence of the chromosomes, but there is also evidence of apparent chromosomal rearrangements as well as a 400 kb insertion in the Rose City isolate relative to the Fl1 isolate chromosome 5.

**Figure 5:**
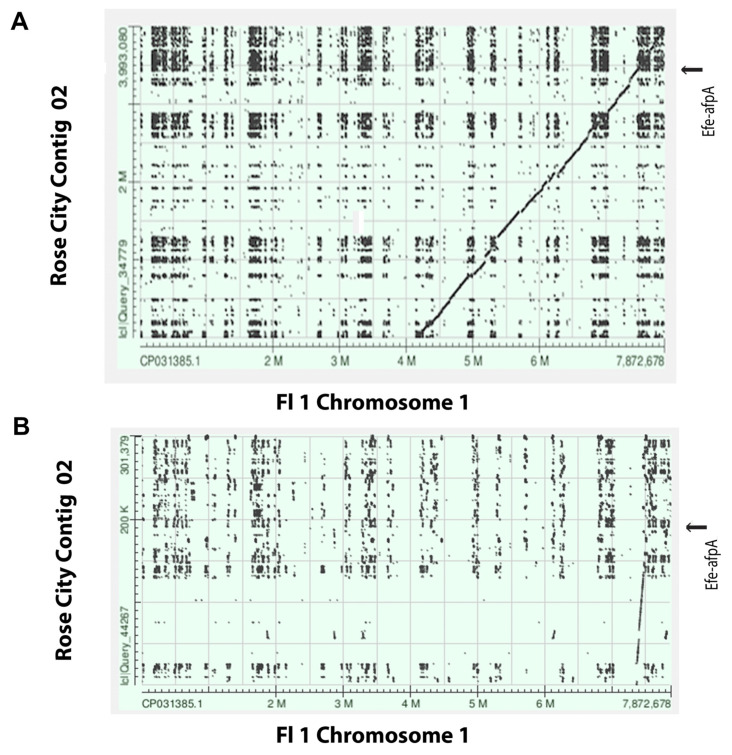
(**A**). Screen shot of dot plot comparison of the *E. festucae* Rose City isolate PacBio contig 02 sequence with the Fl1 chromosome 1 sequence. (**B**). Screenshot of the dot plot region of Rose City contig 02 sequence that contains *Efe-afpA* with the Fl1 chromosome 1 sequence. The arrows indicate the position of *Efe-afpA* in the Rose City sequence.

**Figure 6:**
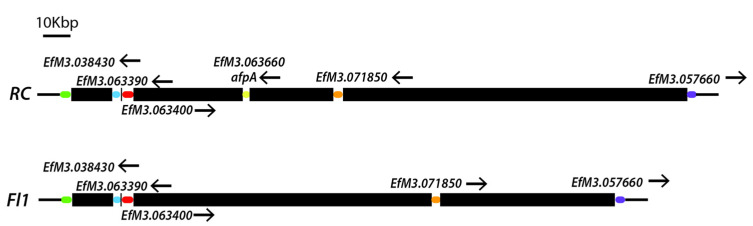
Diagrams from the *E. festucae* Rose City (RC) isolate contig 2 region containing *Efe-afpA* and the corresponding region from *E. festucae* Fl1 isolate chromosome 1 (GenBank accession CP031385.1). Corresponding genes from the two isolates are indicated with the same colors and arrows indicate the directions of the coding sequences. The black bars indicate the AT-rich repeated sequences between genes.

*Efe-afpA* was localized on the Rose City isolate contig 02, which corresponded to part of chromosome 1 of the Fl1 isolate. The *Efe-afaA* gene in the Rose City isolate is located in a relative position near the end of chromosome 1 of the Fl1 isolate (Figure 5). Genes surrounding *Efe-afpA* in the Rose City isolate were syntenous with those genes in the Fl1 isolate, which lacks a gene homologous to *Efe-afpA*. As shown in the comparison of the *Efe-afpA* region of the two *E. festucae* isolates (Figure 6), the genes surrounding *Efe-afpA* are conserved but the length of the AT-rich regions between the genes varied between the two isolates and the direction of the coding sequences of EfM3.071850 is opposite between the two isolates.

## 4. Discussion

Here, we successfully applied CRISPR-Cas9 technology to the non-model fungus *E. festucae*. The CRISPR-Cas9 approach was successful in generating knockouts of a gene that was not feasible using the conventional method of homologous recombination. This was likely due to lack of long enough unique flanking regions required for homologous recombination.

The CRISPR-Cas9 gene editing approach that has been developed for eukaryotic systems requires expression of a functional *cas9* endonuclease gene and of a sgRNA, which targets the desired site of editing. The *cas9* gene was originally identified from the bacterium *Streptococcus pyogenes* [30] and was codon-optimized for expression in human cells [9]. For use in eukaryotes a nuclear localization signal is required to direct the Cas9 protein to the nucleus. The 7-amino acid SV40 large T antigen nuclear localization signal [31] is often used, appended to the C-terminus of the *cas9* coding sequence. The human codon-optimized *cas9* gene has been successfully used in several fungal species, including *Saccharomyces cerevisiae* [32], *Neurospora crassa* [33], *Aspergillus fumigatus* [17,34], and *Penicillium chrysogenum* [35]. However, the same construct that was successfully used in *A. fumigatus* [17] was not successful in *E. festucae*. The human codon-optimized *cas9* gene was reported to not be effective in *Trichoderma reesei* [18]. The construct that was successful in generating the *Efe-afpA* knockouts included a *Trichoderma reesei* codon-optimized *cas9* sequence with *T. reesei* pyruvate decarboxylase promoter and terminator sequences.

In addition to a codon optimized *cas9* gene, another key component for generating gene knockouts is sgRNA, whose expression is generally driven by a polymerase III promoter. In the unsuccessful construct, the sgRNA was part of the *cas9* containing plasmid and its expression was driven by the *S. cerevisiae* snRNA52 promoter, a RNA polymerase III promoter. The snRNA52 promoter was successful in generating the polyketide synthase gene *pksP* sgRNA in *A. fumigatus* [17]. However, the construct containing the human codon-optimized *cas9* gene and the snRNA52 promoter for sgRNA expression was not successful in *E. festucae*. Another commonly used promoter for sgRNA is the endogenous U6 promoter. However, the U6 promoter in many fungi has not been identified because the fungal U6 gene often has multiple introns [36]. The U6 promoter of *Epichloë* species has not been identified. Common fungal RNA polymerase II promoters such as *trpC* [37] and *gpdA* [38] have been used in fungal systems to drive expression of sgRNA. However, Arazoe et al. [37] showed significantly less transformation efficiency with the *trpC* promoter than with the endogenous U6 promoter. A simpler alternative to in vivo expression of sgRNA is co-transformation of a plasmid with the *cas9* gene with in vitro transcribed gRNAs as used by Liu et al. [18] in *T. reesei*. This approach was used successfully here in *E. festucae*.

The CRISPR-Cas9 approach to gene knockouts generally results in small indels in the gene of interest, which typically produce a change in reading frame and an early termination codon. One of the *Efe-afpA* knockouts recovered here was of this type with a single base pair insert. The other knockout had a 10 bp deletion of *Efe-afpA* and a 1625 bp insertion at the target site that originated from the transforming plasmid. Similar vector insertions at the target site were reported in *A. fumigatus* [17], *Nodulisporium* sp. [39], and *Sclerotinia sclerotiorum* [40].

The *Efe-afpA* knockouts were generated with the aim of inoculating them into strong creeping red fescue in order to determine if absence of the antifungal protein gene in endophyte-infected plants leads to disease susceptibility. However, the *Efe-afpA* knockout isolates could not be inoculated into strong creeping red fescue seedlings. In contrast, the wild type Rose City isolate and the complemented knockout isolates were successfully inoculated into strong creeping red fescue seedlings. Similar evidence has been used to identify some other *E. festucae* genes as required for the symbiotic interaction. Knockouts of two histone methyltransferases, EfM3.042710 and EfM3.062280, were unable to infect the host plant perennial ryegrass but the complemented knockout isolates were able to infect [41]. The authors proposed that the activity of the histone methyltransferases controlled the expression of genes encoding infection factors. In this study, the inability of the knockout isolates to infect the host grass precluded an evaluation of the lack of *Efe-afpA* on disease resistance or susceptibility of the host.

These results presented here suggest *Efe-afpA* may have a role in the symbiosis, in addition to having antifungal activity. The interaction of fungal plant pathogens and symbionts with their hosts involves effector proteins, characterized as small-secreted proteins that can be important for colonization or for evasion of host defenses [42,43,44]. In general, fungal effector proteins interact with a host plant protein either in the apoplastic space or inside the plant cell and interfere with a host plant function [45]. *E. festucae* expresses numerous small-secreted proteins that may function as effectors in its interaction with the host grass [7]. However, none of these small-secreted proteins have been functionally confirmed as effectors. Hassing et al. [28] analyzed the *E. festucae* genome sequence for potential effectors and identified 141 candidate genes, one of which was *Efe-afpA*. The *E. festucae* antifungal protein has the characteristics of an effector protein in that it is a cysteine-rich small secreted protein and its expression is considerably higher in the infected plant tissue than in culture [28,46]. In addition to the quantitative PCR data presented here, *Efe-afpA* was among the most abundant fungal transcripts in transcriptome studies of *E. festucae* infected leaf sheath and inflorescence tissues [7,47]. The inoculation data presented here suggests that *Efe-*AfpA may be an effector protein required for the symbiotic interaction of *E. festucae* and its host grass strong creeping red fescue. However, if *Efe-*AfpA is an effector protein it must be specific to certain interactions, since most *Epichloë* spp. do not have such a gene in their genomes [6].

In addition to its antifungal activity and the potential that it may be an effector protein, *Efe-AfpA* is interesting from an evolutionary point of view, since most *Epichloë* spp. do not have a similar gene. Is the distribution of *Efe-afpA* among *Epichloë* spp. due to gene loss or gene gain? Phylogenetic analysis of nonhybrid *Epichloë* spp. did not reveal a clear lineage that could be proposed as a point of gene gain or gene loss [6]. The antifungal protein gene in the basal lineage, *E. inebrians*, was more similar to a gene in *Pochonia chlamydosporia*, a nematode egg parasitic fungus, than to the antifungal protein genes in the more derived lineages of *E. festucae*, *E. baconii*, and *E. aotearoae* suggesting horizontal gene transfer from *Po. chlamydosporia* to *E. inebrians* [6]. Additional research will be required to determine the origin of the patchy distribution of antifungal protein genes among *Epichloë* spp.

Analysis of the *E. festucae* Fl1 genome revealed the genome is structured such that transposon rich AT repeat blocks largely lacking genes are interspersed with gene-rich regions that are largely repeat free [29]. The authors proposed that genes within such fast-evolving AT-rich blocks may contribute to strain adaptation leading to such regions hosting lineage-specific genes. *Efe-afpA* is a single gene located within an AT-rich block and is lineage specific. The long read sequence data generated here to determine the genomic context of *Efe-afpA* will also be a resource for future studies on genome evolution among *E. festucae* isolates.

In summary, here we have demonstrated the utility of the CRISPR-Cas9 approach to gene deletion in *Epichloë*. For *Efe-afpA*, this approach was critical since the standard approach of homologous recombination was not possible due to the lack of long 5′ and 3′ unique regions required for recombination. Unexpectedly, both of the independent **Δ***Efe*-*afpA* isolates were unable to infect host plant seedlings, although 3 of the 4 complemented knockout isolates were able to infect. Based on these results our hypothesis is that in addition to having antifungal activity, *Efe-AfpA* may be an effector protein required for infection and the symbiosis of *E. festucae* with its host grass strong creeping red fescue. Additional research will be required to determine if *Efe-AfpA* is indeed an effector protein.

## Figures and Tables

**Table 1:** Results of inoculating strong creeping red fescue (*Festuca rubra* subsp. *rubra*) seedlings with the *Epichloë festucae* wild type Rose City (RC) isolate, the two Δ*Efe-afpA* knockout isolates, and the four Δ*Efe-afpA/Efe-afpA* complemented isolates.

	Not Infected	Infected
Wild type RC	97	8
Δ*Efe-afpA*(1a-7t8s3)	180	0
Δ*Efe-afpA* (1c-3s5)	180	0
Δ*Efe-afpA/Efe-afpA*(1a-7t8s3-B)	74	0
Δ*Efe-afpA/Efe-afpA*(1a-7t8s3-I)	64	1
Δ*Efe-afpA/Efe-afpA*(1c-3s5-X)	50	2
Δ*Efe-afpA/Efe-afpA*(1c-3s5-L)	54	2

**Table 2:** *Epichloë festucae* Rose City isolate long-read genome assemble.

Total Contigs	46
Total bases	37,116,035
Max contig length	4,765,072
N50 length	2,773,382

## Data Availability

The data presented in this study are openly available at DDBJ/ENA/GenBank under the accession JADWOS000000000.

## Supplementary Materials

The following are available online at https://www.mdpi.com/2076-2607/9/1/140/s1, Figure S1: Sequence alignment of mutated *Efe-afpA* region of wild type isolate Rose City and Δ*Efe-afpA* knockout isolate 1c-3s5, Table S1: Sequences of oligonucleotide primers used in this study.
