# Supplementary material for: The Epichloë festucae Antifungal Protein Efe-AfpA Is also a Possible Effector Protein Required for the Interaction of the Fungus with Its Host Grass Festuca rubra subsp. rubra
Source: Microorganisms. 2021 Jan 9;9(1):140. doi: 10.3390/microorganisms9010140 (PMC7827515; doi:10.3390/microorganisms9010140)
Supplement: Supplementary file 1 [file microorganisms-09-00140-s001.zip › microorganisms-1044856-supplementary/Supplementary Figure S1.docx]

Wild type ATGCAAATCACCGTGGTCGCGGTTTTCCTCCTCTCTGCAATGGGCGGAGT

1c-3s5 ATGCAAATCACCGTGGTCGCGGTTTTCCTCCTCTCTGCAATGGGCGGAGT

AGCCACACCCATCAACTCCAGAATCAATCCTGTTGATGCCAGGGCGGAGACTGGCATTCT

AGCCACACCCATCAACTCCAGAATCAATCCTGTTGATGCCAGGGCGGAGACTGGCATTC-

GATCACGTA–––––––––––––––––––––––––––––––––––––––––––––––––––

–––––––––TACTTAGGGGAAATAAAGGTTCTTGGATGGGAAGATGAATATACTGAAGAT

––––––––––––––––––––––––––––––––––––––––––––––––––––––––––––

GGGAAAAGAAAGAGAAAAGAAAAGAGCAGCTGGTGGGGAGAGCAGGAAAATATGGCAACA

––––––––––––––––––––––––––––––––––––––––––––––––––––––––––––

AATGTTGGACTGACGCAACGACCTTGTCAACCCCGCCGACACACCGGGCGGACAGACGGG

––––––––––––––––––––––––––––––––––––––––––––––––––––––––––––

GCAAAGCTGCCTACCAGGGACTGAGGGACCTCAGCAGGTCGAGTGCAGAGCACCGGATGG

––––––––––––––––––––––––––––––––––––––––––––––––––––––––––––

GTCGACTGCCAGCTTGTGTTCCCGGTCTGCGCCGCTGGCCAGCTCCTGAGCGGCCTTTCC

––––––––––––––––––––––––––––––––––––––––––––––––––––––––––––

GGTTTCATACACCGGGCAAAGCAGGAGAGGCACGATATTTGGACGCCCTACAGATGCCGG

––––––––––––––––––––––––––––––––––––––––––––––––––––––––––––

ATGGGCCAATTAGGGAGCTTACGCGCCGGGTACTCGCTCTACCTACTTCGGAGAAGGTAC

––––––––––––––––––––––––––––––––––––––––––––––––––––––––––––

TATCTCGTGAATCTTTTACCAGATCGGAAGCAATTGGACTTCTGTACCTAGGTTAATGGC

––––––––––––––––––––––––––––––––––––––––––––––––––––––––––––

ATGCTATTTCGCCGACGGCTATACACCCCTGGCTTCACATTCTCCTTCGCTTACTGCCGG

––––––––––––––––––––––––––––––––––––––––––––––––––––––––––––

TGATTCGATGAAGCTCCATATTCTCCGATGATGCAATAGATTCTTGGTCAACGAGGGGCA

––––––––––––––––––––––––––––––––––––––––––––––––––––––––––––

CACCAGCCTTTCCACTTCAGAAGCCGCAGGTGTCGAGCCGGGAGGAGTTTTCGCAGTGGC

––––––––––––––––––––––––––––––––––––––––––––––––––––––––––––

TGTGCCGCGCGCACAACGATGTCAACCGGAAGCTGGGCAAGCCCGAGTTTGACTGCTCGC

––––––––––––––––––––––––––––––––––––––––––––––––––––––––––––

GGGTGGACGAGCGGTGGAGGACGGGGTGGAAGGACGGTCGGTGCGACTGATTGGCCATGA

––––––––––––––––––––––––––––––––––––––––––––––––––––––––––––

ATTCCATCTTTCGAGGACGGACGAAGATACTGTACGATTAATGAAAGGAGGGAGCATTCT

––––––––––––––––––––––––––––––––––––––––––––––––––––––––––––

TCGACTTGCGGCAATTGCATGCACATGTACGATTGGAAGCGCGGGCGATGTATTCGCAAT

––––––––––––––––––––––––––––––––––––––––––––––––––––––––––––

CATGTTTAGAAGGACGGCGTTTGGAAACGTTGGGATGCTGTTGAAGCGTTGGAAACAGGG

––––––––––––––––––––––––––––––––––––––––––––––––––––––––––––

GCAATTAGAAACACCGAGCCAGACAGAGTCAATGGTACGAGGTCAGCCAGTATCATGACC

––––––––––––––––––––––––––––––––––––––––––––––––––––––––––––

TGTGTGCGCATGGTGGCGAGAGATTCCGAGCCATGCCACGGGAGACGAGCAATGAAAAAA

––––––––––––––––––––––––––––––––––––––––––––––––––––––––––––

CTCTTCACTCACTTGTCGAGGCTCTCTCAACCTATCGACTTATCAAGTAGACGATGAAAG

––––––––––––––––––––––––––––––––––––––––––––––––––––––––––––

CCTTGCAACTGTGGTGATGTGGCTCATCAATGTGCGACGTCGTATCCATGTCTGAGGCCA

––––––––––––––––––––––––––––––––––––––––––––––––––––––––––––

TTCGATATCGTGATGCGACTACCTAGTAAAGCCCGGCCAGAGGGCAAACCGGGGCGACAG

––––––––––––––––––––––––––––––––––––––––––––––––––––––––––––

GGGCAGGCAATTGACCGGATGGCTGCATGTGCCGAAGCAGCCCCGATGGAATCGAGATGT

––––––––––––––––––––––––––––––––––––––––––––––––––––––––––––

CTGTCGGATGGACCGCTGAGCGGCCTGGCAAGGTGTCCCAGATACGAAGATGGAAGTGAA

––––––––––––––––––––––––––––––––––––––––––––––––––––––––––––

GTCAGAGGTGGTCGTTAATTGTCCGACGAGCGAATCGGCCGCTCCTTCGGATTGCCGGCT

––––––––––––––––––––––––––––––––––––––––––––––––––––––––––––

CTGCTGTATGTACCGTGCATGAAGCCACCCGGGATCCATGTTACGATGGATAGGTTCCAA

––––––––––––––––––––––––––––––––––––––––––––––––––––––––––––

CTCTCTAGTAGCTATAGTGGACCTGAGGCTATCTAGTATCACTGGAGGAGCAGCCGTCCA

––––––––––––––––––––––––––––––––––––––––––––––––––––––––––––

CTATCGTCGAGCGCTGTAGAAGCAGCTGCATTAGCGGCTGCCCACCCGCGCAGAAATGGC

––––––––––––––TGAAGGAGTGAGTTGATTTTGTGTCACAATACCCGATTACTCTGAG

CCCATTACATCACTTGAAGGAGTGAGTTGATTTTGTGTCACAATACCCGATTACTCTGAG

TATTCAATAACCACGGAGTCCCCTCTATAAAGACATGTTCTAGAGCCAAAAACGAATGCA

TATTCAATAACCACGGAGTCCCCTCTATAAAGACATGTTCTAGAGCCAAAAACGAATGCA

AGTACAAAAATCAGAACAACAAGGACACGTTCGTCAAGTGCCCGTCGTTCGCAAACAAGA

AGTACAAAAATCAGAACAACAAGGACACGTTCGTCAAGTGCCCGTCGTTCGCAAACAAGA

AGGTAAAGGTTCTTTTTTGCCCCCTTTTGAGGCTGCCTATTTCCAAGCTTACGTCTGATT

AGGTAAAGGTTCTTTTTTGCCCCCTTTTGAGGCTGCCTATTTCCAAGCTTACGTCTGATT

AATCCACCGTTACAGTGCACCAAAGACAACGCCAAGTGTAGCTTTGACAGTTATTCTCGA

AATCCACCGTTACAGTGCACCAAAGACAACGCCAAGTGTAGCTTTGACAGTTATTCTCGA

GCTGTCACGTGTCATTAG

GCTGTCACGTGTCATTAG

Supplementary Figure S1. Sequence alignment of mutated *Efe-afpA* region of wild type isolate Rose City and **Δ***Efe-afpA* knockout isolate 1c-3s5. A 1625 bp insertion in 1c-3s5, highlighted in grey, is a large fragment from the transformation vector G4. Target guide RNA sequence and PAM designed for CRISPR-Cas9 are highlighted in green and red, respectively.
